# Neuroinformatics: From Bioinformatics to Databasing the Brain

**DOI:** 10.4137/bbi.s540

**Published:** 2008-05-14

**Authors:** Thomas M. Morse

**Affiliations:** Department of Neurobiology, Yale University School of Medicine, 336 Cedar Street, New Haven CT 06510

**Keywords:** comparison, similarity, difference, overlap, understand, between

## Abstract

Neuroinformatics seeks to create and maintain web-accessible databases of experimental and computational data, together with innovative software tools, essential for understanding the nervous system in its normal function and in neurological disorders. Neuroinformatics includes traditional bioinformatics of gene and protein sequences in the brain; atlases of brain anatomy and localization of genes and proteins; imaging of brain cells; brain imaging by positron emission tomography (PET), functional magnetic resonance imaging (fMRI), electroencephalography (EEG), magnetoencephalography (MEG) and other methods; many electrophysiological recording methods; and clinical neurological data, among others. Building neuroinformatics databases and tools presents difficult challenges because they span a wide range of spatial scales and types of data stored and analyzed. Traditional bioinformatics, by comparison, focuses primarily on genomic and proteomic data (which of course also presents difficult challenges). Much of bioinformatics analysis focus on sequences (DNA, RNA, and protein molecules), as the type of data that are stored, compared, and sometimes modeled. Bioinformatics is undergoing explosive growth with the addition, for example, of databases that catalog interactions between proteins, of databases that track the evolution of genes, and of systems biology databases which contain models of all aspects of organisms. This commentary briefly reviews neuroinformatics with clarification of its relationship to traditional and modern bioinformatics.

## Introduction to Neuroinformatics

Neuroinformatics as a field that includes building databases and tools for understanding the nervous system was initiated in the early 1990s (Huerta et al. 1993). During the subsequent decade development started on dealing with the complex types of data that characterize studies of the nervous system. In 2000, a paper suggested that web portals (databases) were needed to catalog the burgeoning number of databases and tools (Smaglik, 2000). Around 2004, a database (SfN (Society for Neuroscience) Neuroscience Database Gateway 2007) (this web link and others mentioned in this article are provided in the references) emerged to satisfy this need, and a more ambitious implementation of the approach (Neuroscience Information Framework 2007) is currently under development.

It may facilitate understanding the complexity of neuroinformatics by first discussing a related field, bioinformatics. The first protein data collections were made in the late 1970’s; reviewed in (Ouzounis and Valencia, 2003; Smith, 1990). Investigators realized that the computer was an essential tool to keep track of either the series of letters (e.g. GCAT) that represented the base sequence that makes up the nucleic acids or the sequence of amino acids that make up proteins (Ouzounis and Valencia, 2003; Smith, 1990; Dayhoff, 1978; Bernstein, et al. 1977). Databases for genes, European Molecular Biology laboratory’s EMBL-Bank and the National Center for Biotechnology Information’s GenBank, were launched in the early 1980s (National Library of Medicine 2005a; National Library of Medicine 2005b).

### Neuroinformatics compared to traditional bioinformatics

Traditional bioinformatics is the field that encompasses comparing and databasing the genome (DNA), and related molecules (RNA, proteins), and also modeling the structure and function of existing and new (designed) proteins. We use the term traditional bioinformatics to acknowledge that the explosion of activity in bioinformatics has grown beyond the origin of bioinformatics (further discussed below). Bioinformatics can be defined most generally (although not all investigators choose to do so) as all combinations of biology (life sciences) and informatics (computer and statistical methods). Bioinformatics most general definition would then include neuroinformatics as well as systems biology (that seeks to model all aspects of life) as part of bioinformatics. Several online descriptions further elaborate or nuance this broad view of bioinformatics (Wikipedia contributors 2008), (Altman, 2006), (Huerta et al. 2000). Bioinformatics’ original focus was very specific, profound, and important, indeed, most articles in this Journal fall within the original discipline. We return to elaborating traditional bioinformatics since it is helpful to understand neuroinformatics (and other recent developments in bioinformatics). In early bioinformatics the essential data is the list of letters that make up the sequence. Other example attributes are the species that the sequence is from, chromosomes (if the sequence is DNA) that the sequence belongs to, and the name(s) of the sequence. It was critical to develop tools to allow comparisons between genes, thereby allowing statements to be made about how similar the genes are across alleles, related genes (most genes are thought to be formed from other ancestral genes by duplication and subsequent mutation) and across species. Traditional bioinformatics is comparatively simpler than the newer extensions to bioinformatics such as neuroinformatics because the basic data type in traditional bioinformatics is the sequence. Conceptually traditional bioinformatics consists of sequence oriented databases plus tools to search (on one database, or across databases), to compare (either on-line databases or download-able software), and increasingly to model the molecules related to the sequences.

In contrast, neuroscience data is diverse and heterogeneous. In each subfield of neuroscience, however, there is often an associated primary type of data and neuroinformatics tools to store in databases, to search, to compare, and increasingly to model the physical system. The cross-disciplinary nature of neuroinformatics has required collaboration of teams of scientists with mapping efforts and/or hypothesis-driven goals. Cultural issues (funding, peer-review of journal articles, and promotion reviews of scientists) are present as a result of these efforts that are new (but not restricted to neuroinformatics) (Insel et al. 2003).

## Neuroinformatics Overview

This section discusses the range of experimental and theoretical neuroinformatics topics. For further information, the online resources can be consulted (Neuroscience Database Gateway 2007) and recent publications (Bjaalie and Grillner 2007; French and Pavlidis 2007).

Neuroinformatics experimental databases may store function and anatomy annotations of nervous system genes, images (acquired with different methods such as structural and functional magnetic resonance imaging (MRI and fMRI), tissue staining at spatial scales from subcellular electron microscope images to tens of centimeters slices though brains of monkeys, optical recordings of voltage and chemical activated dyes, etc.), and atlases of central and peripheral nervous systems (Neuroscience Database Gateway 2007). Several projects tackled the difficult problem of open ended data-sharing with heterogeneous data (Gardner et al. 2001; Lam et al. 2007; Marenco et al. 2004; Martone et al. 2004). Experimental and descriptive data, in the open ended case, is documented with meta-data which describes its content and format for successful sharing. Other projects attempted to advance new experimental methods (wavelet analysis in fMRI (Fadili and Bullmore, 2002)) and data analysis tools for MRI mapping of brains to surfaces for comparison between brains from the same species (in humans this has medical applications (see later)), and also comparisons between different species nervous systems (Dickson et al. 2001; Mazziotta et al. 2001; Toga et al. 2006; van Essen et al. 1994). Further tools allow the recognition of objects within the fMRI data-sets, improve the quality of fMRI images and systems to store and share fMRI data-sets (Bullmore et al. 2001; Kennedy DN Haselgrove C, 2006). Other projects were designed to setup databases of in-situ hybridization data to provide a permanent accessible archive for resources in danger of disappearing (much of this data exists in slides in individual investigators laboratories) (Interactive yMultiple Gene Expression Maps 2008; Rosen et al. 2003). Several projects created online atlases and maps of human, macaque (monkey), rat, and mouse brains (see databases having atlases as categories in SfN Neuroscience Database Gateway 2007). Several projects combined databases of images with annotations of gene-expression (within the images) (Rosen et al. 2003; Martone et al. 2004; Interactive Multiple Gene Expression Maps, 2008).

Theoretical neuroinformatics projects tackled diverse topics: modeling cortical maps (Bednar et al. 2004), modeling the olfactory bulb (Migliore et al. 2005) and storing computational neuroscience models in a web accessible database (Peterson et al. 1996), developing new wavelet based and source separation analysis tools for fMRI data (Fadili and Bullmore, 2002), detailed tools for neuromorphological modeling (Ascoli et al. 2001), automatic cortical surface reconstruction mapping and tools for specifying a 2-D coordinate system for the mapping (Dale et al. 2000), and creating realistic computational models capable of predicting human auditory responses to a wide range of acoustic stimuli including acoustic trauma (Hubbard and Mountain, 2006).

In conclusion, there is a wide variety of neuroscience databases and tools that support neuroscience and biomedicine. The field of neuroinformatics might be perceived as an extended and elaborated application of analogous tools to those found earlier to be critical for the development of bioinformatics, but applied to a broader, heterogeneous types of data at many levels of function. The depth and breadth of neuroscience information is beginning to be organized through databases of databases. These, and the information in the databases they contain, will aid searching in, comparing, and modeling nervous systems at spatial scales from the level of molecules to behavior, in normal, diseased, or injured humans and animals. Neuroinformatics is becoming essential to neuroscience investigators and clinicians for conducting scientific inquiry, and practicing medicine in our time of rapidly expanding cross-disciplinary knowledge.

## Neuroinformatics Examples: Multiple Database Search

### Using the national center for biotechnology information (NCBI)

**Figure 1:**
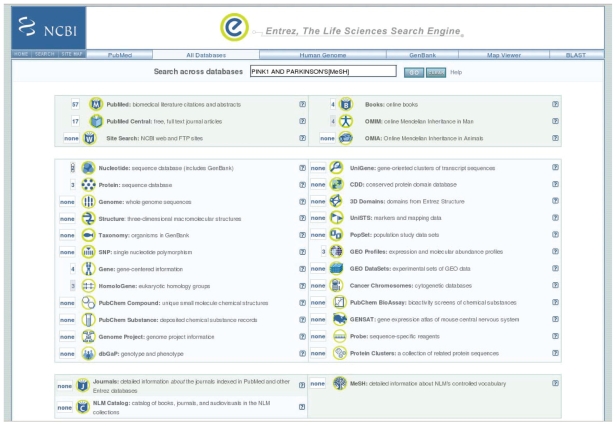
All database search in PubMed. A collection of 23 databases are searched simultaneously for a boolean expression of keywords of interest. Restricting searches with MeSH terms targets the results to relevant topics.

A neuroscience search that demonstrates an overlap in traditional bioinformatics and neuroinformatics starts with an “All database” search at PubMed (National Center for Biotechnology Information 2007). This powerful search engine simultaneously searches through 28 databases which includes entries in the diverse topics of literature (articles in journals and books), sequence databases, metadata (descriptions of the data or format in which the data is stored), databases on Journals (themselves, not the articles in them) and vocabulary, and bibliographic data for the National Library of Medicine holdings of books, software, and other resources (Fig. 1). This is useful to simultaneously find information about genes and also the literature that describes findings about the gene. A search on the Parkinson’s related gene PINK1 results in 114 articles found in PubMed, however using the Medical Subject Heading (MeSH) (National Library of Medicine 2007) terms (selected “meaningful words”, i.e. words whose definitions are precisely specified, the search “PINK1 AND PARKINSON’S[MeSH]” finds 67 articles which are then known to be relevant to PARKINSON’S disease (Fig. 1). The MeSH terms help the user find the items in the databases that have the right context, e.g. the Nucleotide sequence database (includes GenBank) finds 41 hits in the unrestricted PINK1 search, however, when the PARKINSON’S term is added the number of hits drops to 2 and the reports associated with the entries are targeted to Parkinson’s clinical research. This example demonstrated how targeted searches through a collection of databases produces results that are of immediate interest to the neuroscience investigator.

### Using the neuroscience database gateway (NDG)

**Figure 2:**
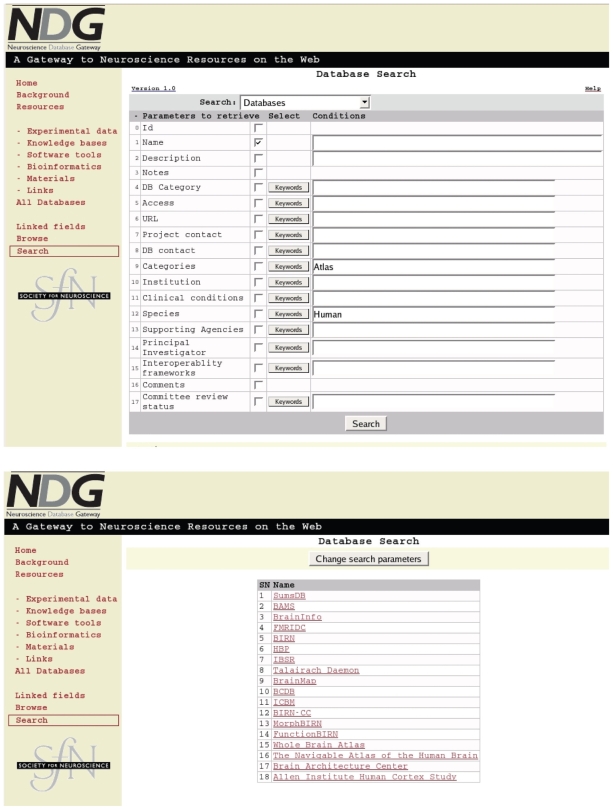
Database search in NDG. The SfN Neuroscience Database Gateway in a sample search for Human Atlases (top). There were 18 results (bottom) of which numbers 15 and 16 (by their names) are suitable for browsing to gain human brain familiarity.

Let us imagine that we are an investigator looking for web sites that contain human brain atlases to reexamine some facet of nervous system anatomy. Starting at the NDG (SfN Neuroscience Database Gateway 2007) home page click on the “Search” link in the left hand column and enter “Atlas” under categories and “Human” under species and press the “Search” button (Fig. 2 top). If the search terms are not known the “keyword” buttons for each field can be pressed which pop-up a window where multiple search terms can be selected. In the results (Fig. 2 bottom) numbers 15, “Whole Brain Atlas”, and 16, “The Navigable Atlas of the Human Brain”, are seen by their names to be immediately relevant. The reader is invited to further explore.

## Neuroinfomatics Examples: Representative Human Brain Projects (HBP)

To give the reader a flavor of the types of databases that are being used in neuroinformatics, we provide a brief orientation to selected projects supported by the Human Brain Project (National Institute of Mental Health 2007). The selections are intended to sample a small number of representative topics; for orientation to the broad scope of current neuronformatics databases, the reader is referred to the Society for Neuroscience (SfN Neuroscience Database Gateway 2007).

**Figure 3:**
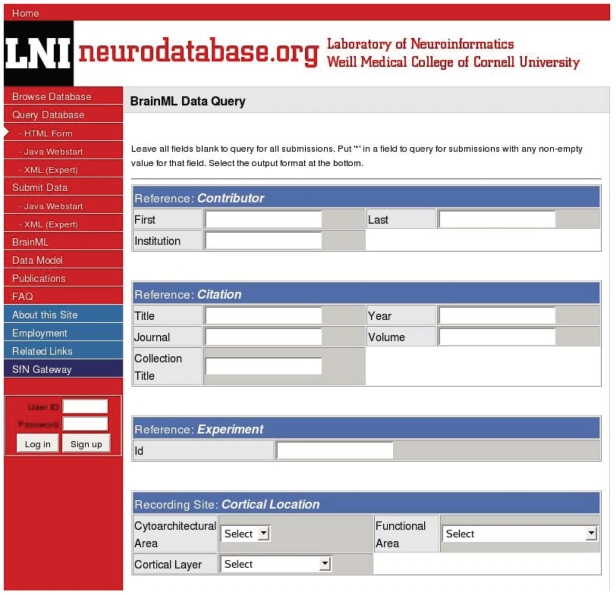
Searching for entries at Neurodatabase.org. This is a pilot project to explore ways in which electrophysiological data can be stored and then retrieved. Annotated data includes electrical recordings from monkey brains, with simultaneously acquired video and behavioral activity time series. Image copyright Weill Cornell medical College, used by permission.

Managing data acquired in the laboratory is challenging. It is hoped that the difficulties of organizing data before, and preserving it after publication, can be ameliorated by storing it in online databases. This also makes it easier to share the data with colleagues who may want to reanalyze the data to verify the hypotheses, or look for support for other (perhaps unrelated) hypotheses. There are a few neuroinformatics projects that are designing methods to store physiological datasets in web-databases, one of which, “Databases and data models enabling neuroinformatics” headed by Daniel Gardner (Laboratory of Neuroinformatics Weill Medical College of Cornell University 2007a) (Fig. 3).

The group is developing protocols in XML that can be used to make the data self-describing, i.e. when the data is encapsulated in these XML files, the description of what the data is, is included along with the data itself. Gardner’s group has made important contributions to the conceptual framework for classifying all neurophysiology data (see the Common Data Model in (Gardner et al. 2001)), and are developing an exchange format called BrainML (Laboratory of Neuroinformatics Weill Medical College of Cornell University 2007b) In their prototype database (Fig. 3) the primary data are electrical recordings and behavioral event time series; other attributes are recording sites, associated publications, brain region and recording sites within that region, type of neuron, species of animal, etc. They initially choose to store types of data that were of immediate use by an electrophysiologist colleague of theirs who made electrical recordings from pyramidal cells in somatosensory and related cortical areas that were correlated with video-taped behavioral events during a reaching task in macaque. This provided a prototype system which demonstrated methods to store annotated physiological data. Their group has recently increased the scope of the neurophysiology data that’s web accessible. The new data includes electrical recordings from retinal ganglion cells, marmoset lateral geniculate nucleus, and macaque primary visual and inferior temporal cortex.

**Figure 4:**
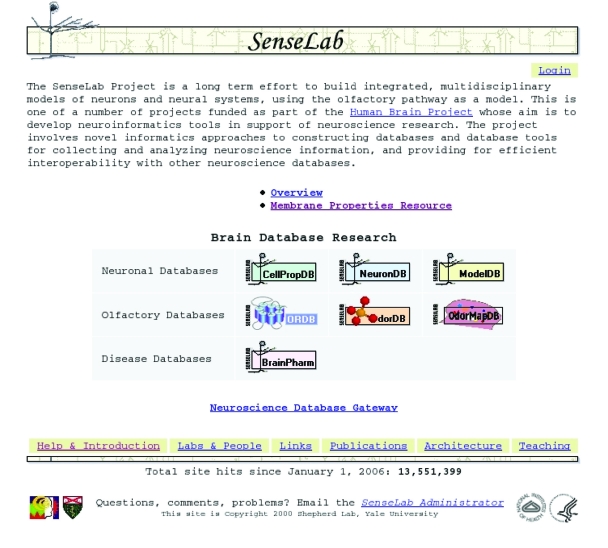
Senselab. A collection of databases that explore pilot projects in several subfields in Neuroscience: olfactory receptor genes, computational neuroscience models, neuronal membrane properties and olfactory maps. See text for more details. Image used by permission.

Many neuroinformatics projects are multidisciplinary. “SenseLab: Integration of multidisciplinary sensory data”, directed by Gordon Shepherd at Yale University in New Haven Connecticut, has developed a collection of pilot databases and informatics technologies (Shepherd Laboratory Yale University 2007) (Fig. 4).

SenseLab is implemented in a relational database framework called Entity, Attribute, Value, with Class and Relationships (EAV/CR) that allows for the structure of the database to be altered (creating new tables, or new columns within a table) without having to rebuild the database or reprogram the various interfaces. The group has developed convenient database-building web tools and continues to extend and develop EAV/CR into new technologies (applications in XML and web-form libraries) (Marenco et al. 2003). An Olfactory Receptor genes database, ORDB, stores the sequences that make up the olfactory receptor proteins (Crasto et al. 2002) and an odor functional maps database, OdorMapDB, supports archiving, searching, and comparing images of olfactory bulb activity in response to odor presentations (Liu et al. 2004). Databases on the properties of neurons, CellPropDB, and neuronal compartments, NeuronDB, store information on the neuronal and compartment distribution of membrane proteins—ion channels and receptors—and neurotransmitters which are known to be present (or known to be absent) from a particular neuron or neuronal compartment. This information is needed by both electrophysiologists, who are searching for information relevant to their experiments, and modelers seeking to construct a biologically realistic model of a particular neuron’s electrical activity, or network of neurons.

A fifth database, ModelDB, stores the computer code for published computational neuroscience (CNS) models (Hines et al. 2004). It is frequently difficult if not impossible to recreate a computer model from a CNS publication due to typos and omissions either in the original article or in the attempt to reproduce the model (Migliore et al. 2003). Storing the computer code either makes it possible or, at least, saves investigators time in recreating the model or extending the model to test new hypotheses (by altering or using the model as a building block or template in a larger model).

In addition to the ion channels and receptors present in neurons, the shape of the neuron (morphology) contributes to their patterns of electrical activity (see for example Mainen and Sejnowski 1996). A group headed by Giorgio Ascoli at George Mason University in Washington DC studies neuronal morphologies with artificially generated neurons. This project called “L-Neuron: generation and description of dendritic morphology” has a web-based archive (Computational Neuroanatomy Group 2007) where both real and artificially generated cell morphologies are available in forms convenient for modelers use (Ascoli et al. 2001).

The artificial cells allow modelers to study the contribution of morphological parameters and also to determine if there is an equivalence between the virtual morphologies and real cells. Part of their project involved the analysis of real cell morphologies influence on electrical behavior for a particular cell type (e.g. Li and Ascoli, 2006).

At the University of California San Diego, Mark Ellisman and Maryann Martone lead a project to store and process detailed images and related cellular data in a project called “The Cell Centered Database” (National Center for Microscopy and Imaging Research 2007). Ongoing projects using one and two color dye injections and immunocytochemistry investigate protein distribution in neurons. The presence or absence of proteins in specific neuronal compartments have been correlated with basic functional neuronal properties such as excitability and information processing underscoring the importance of this work (Martone et al. 2004). Images are acquired with both in-house and remote electron microscopes and confocal microscopy. Another project developed the capability of remote operation of an electron microscope over the internet (Hadida-Hassan et al. 1999).

Neuroinformatics projects involved with either the storage or manipulation of MRI data (12 of the original 37 HBP projects) are the largest representation of an individual topic (MRI). A project run by Michael Gazzaniga and John van Horn, formerly of Dartmouth, now at the University of California-Santa Barbara, entitled “The national fMRI data center”, seeks to provide a repository where all cognitive neuroscience imaging data can be archived and therefore shared (Van Horn et al. 2004). The data center has a relationship with the Journal of Cognitive Neuroscience from which all published articles are required to submit their fMRI data sets to the fMRI data center. This is the first neuroinformatics example of such a requirement, however in the field of bioinformatics an analogous requirement to submit sequence data to online databases for essentially all journals across the field is the status quo, showing the relative maturity of bioinformatics. A PubMed-like search interface at the fMRI data center locates datasets; the datasets are too large to send over the internet so are mailed as DVDs in response to (online) requests. Providing these data sets to the cognitive neuroscience community enables them to assess the original publication, find support for additional hypothesis, and also to evaluate methods.

**Figure 5:**
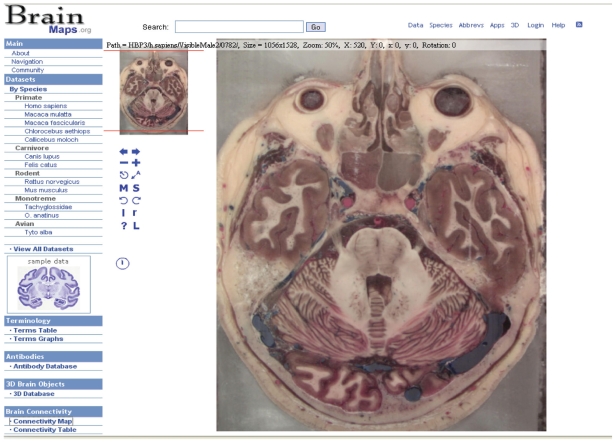
Brainmaps.org has a collection of slides of human and 11 other species that are navigable online. The human brain slice shown here is one of 700 slides (from one individual) stained with the cyro method at 330 microns per pixel. Image used by permission.

Edward Jones directs an informatics project on human and monkey brain atlases (Jones Laboratory University of California Davis 2007) (Fig. 5). The online interface provides views into maps which are derived from MRI and many other staining methods (Mikula et al. 2007). Curating tools are provided for manipulating images so that they can be made to appear with consistent color and with tissue rips repaired. An exciting direction they are working towards is the goal of providing three dimensional virtual reality modeling language code (VRML) and their own method (Trotts et al. 2007) maps of brain regions that allow the viewer to image and travel continuously in a virtual world, thus providing a more intuitive view into the complexity of nervous system anatomy.

**Figure 6:**
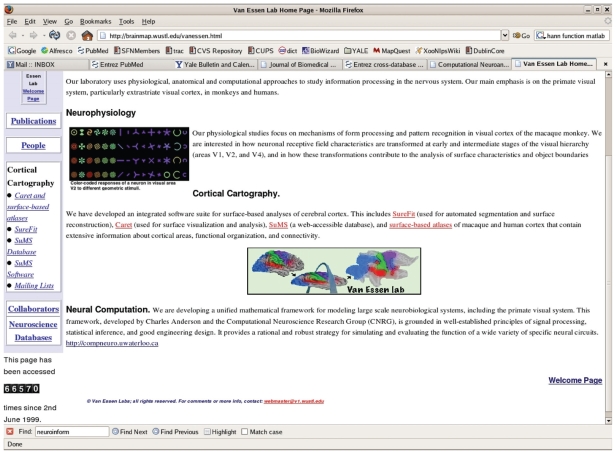
The Van Essen lab is developing tools that transform 3-D cortical maps to 2-D (flat) maps for comparison of functional and structural images between individuals and between species. Image used by permission.

The variability between individuals and larger differences between species provides a challenge for comparisons of brain maps. One response to this challenge has been the creation of methods to transform the cortex onto a flat map where (in two dimensions) comparisons are more manageable. David van Essen is principle investigator on two projects: “Reconstruction and representations of cerebral cortex” (Van Essen Laboratory University of Washington St Louis 2007a) and “Surface Management System” (Van Essen Laboratory University of Washington St Louis 2007b) that store images from monkey and human and make tools available for their comparison (van Essen et al. 1994; Dickson et al. 2001) (Fig. 6).

James Brinkley guides a project, “Structural information framework for brain mapping” (Structural Informatics Group University of Washington Seattle 2007) that takes an alternative approach to brain mapping in its Xbrain project. They relate multiple patient data to spatial or normalized anatomical models. In one of their applications during surgery epilepsy patients have small regions of their brain in the language region numbered. These regions are then mapped with functional tests; stimulating those regions during an object naming task (requesting the patient to name an object) reveals whether or not those stimulations interferes with task. It has previously been shown that if the stimulation interferes with the task then the tissue should not be removed because doing so has a strong correlation with aphasia in postoperative patients. Anatomical correlates of language can be mapped during surgery (Modayur BR et al. 1996). The group continues to prototype methods for three dimensional navigation in brain maps (Poliakov et al. 2005).

**Figure 7:**
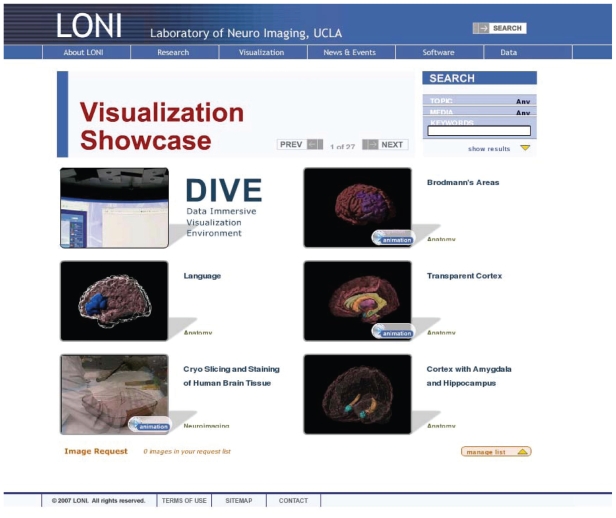
Laboratory of Neuro Imaging (LONI). Over 60 national and international projects to advance image analysis approaches to investigate brain structure and and function in health and disease. Courtesy, Dr. Arthur W. Toga, Laboratory of Neuro Imaging at UCLA.

At University of California Los Angeles, Arthur Toga and John Mazziotta direct diverse projects to store and analyze human brains with 9 different imaging methods including MRI and fMRI. Their laboratory (Laboratory of Neuro Imaging 2007) (Fig. 7) and associates (International Consortium for Brain Mapping 2007), whose goal is the development of a probabilistic reference system for the human brain (Mazziotta et al. 2001), support over 60 national and international collaborations. These efforts include the development of deformation methods to standardize and classify individuals from young to elderly in normal and patients with Alzheimer’s and other diseases (Toga et al. 2006).

## Conclusion

Neuroinformatics is a premier example of the current accelerating exciting growth in bioinformatics knowledge and data. Neuroinformatics databases and tools span a wide range of spatial scales and heterogeneous types of data stored and analyzed. Neuroinformatics databases, together with innovative software tools, are essential for understanding the nervous system in its normal function, in neurological disorders, and have clinical applications. The briefly represented neuroinformatics projects introduced exciting samples of recent developments to the broader bioinformatics community.
